# Qualitative analysis of clinicians’ perspectives on the use of a computerized decision aid in the treatment of psychotic disorders

**DOI:** 10.1186/s12911-020-01251-6

**Published:** 2020-09-17

**Authors:** Lukas O. Roebroek, Jojanneke Bruins, Philippe Delespaul, Albert Boonstra, Stynke Castelein

**Affiliations:** 1 Lentis Psychiatric Institute, Lentis Research, Hereweg 80, 9725 AG Groningen, The Netherlands; 2 University of Groningen, University Medical Centre Groningen, Rob Giel Research Centre, Groningen, The Netherlands; 3 University of Groningen, Faculty of Behavioural and Social Sciences, Groningen, The Netherlands; 4 Maastricht University, Faculty of Psychiatry & Neuropsychology, Maastricht, The Netherlands; 5 Mondriaan Mental Health Trust, Heerlen-Maastricht, The Netherlands; 6 University of Groningen, Faculty of Economics and Business, Groningen, The Netherlands

**Keywords:** Clinical decision aids, Psychotic disorders, Guidelines, Routine outcome monitoring, Clinical decision-making, Shared decision-making, Psychiatry

## Abstract

**Background:**

Clinical decision aids are used in various medical fields to support patients and clinicians when making healthcare decisions. Few attempts have been made to implement such tools in psychiatry. We developed Treatment E-Assist (TREAT); a routine outcome monitoring based computerized clinical decision aid, which generates personalized treatment recommendations in the care of people with psychotic disorders. The aim of this study is to investigate how TREAT is used and evaluated by clinicians and how this tool can be improved.

**Methods:**

Clinicians working with TREAT during a clinical trial were asked to participate in semi-structured interviews. The Unified Theory of Acceptance and Use of Technology (UTAUT) was used as a sensitizing theory to structure a part of the interview questions. The transcripts were analyzed using inductive thematic analysis to uncover the main themes.

**Results:**

Thirteen clinicians (mean age: 49) of which eight psychiatrists and five nurse practitioners, participated in this study. Eight clinicians experienced TREAT as beneficial, whereas five experienced no additional benefits. Thematic analysis revealed five themes surrounding usage and evaluation of TREAT, views on TREAT’s graphic representation of routine outcome monitoring results, guideline based treatment recommendations, contextual factors, effects on patients and effects on shared decision-making. Performance and effort expectancy were perceived as high by clinicians. The facilitating conditions were optimal and perceived social influence was low.

**Conclusion:**

This article presents a qualitative evaluation by clinicians of a computerized clinical decision aid in psychosis care. TREAT was viewed by most clinicians as beneficial during their consultations. The graphic representation of routine outcome monitoring results was well-appreciated and provided input to discuss treatment planning with patients. The treatment recommendations did not change most treatment decisions but supported clinical reasoning. However, some clinicians were unconvinced about TREAT’s benefits. The delivery, applicability and the availability of resources require improvement to increase TREAT’s efficacy. Not all patients responded well to TREAT but the observed facilitation of shared decision-making is promising. All four predictors of the Unified Theory of Acceptance and Use of Technology were positively evaluated by the majority of clinicians.

## Background

### Decision aids

When patients and clinicians draft treatment plans, there are many things to consider. There may be multiple options with no clear, best choice. Clinical decision aids (CDAs) aim to facilitate and improve therapeutic decision-making. They help professionals and patients agree on important treatment options [1]. CDAs do so by assessing needs and providing evidence-based information about treatment options including risks and benefits [1]. Despite the effectiveness of these tools [2], their integration in daily clinical practice remains limited [3, 4]. Less than half of all CDAs are still used after the experimental evaluation period [4]. This is due to a lack of funding or endorsement by organizations, because the tools are out of date or do not fit the existing care processes [4]. Aligning CDAs with guidelines, care standards, clinical policies, existing infrastructure and workflows can augment their uptake within organizations [5]. Often, clinicians remain unconvinced of the benefits of CDAs [6], arguing that they do not agree with their content and use or simply lack time to implement them in their daily clinical practice [4]. Therefore, it is important to involve clinicians in different stages of development of new CDAs and critically evaluate their functionality.

### TREAT

CDAs could benefit psychiatric care, as patients generally have multiple complex care needs, while clinicians often make treatment decisions based on personal preferences [7]. CDAs can reduce the knowledge-gap between available treatments and potential outcomes. They can improve reflection about personal preferences by providing feedback on risk and benefits of specific interventions, for example when deciding on psychiatric medication [2]. Unfortunately, CDAs are poorly implemented and rarely used in psychiatric care. A systematic review of 105 clinical trials of CDAs only included three studies in mental health [2]. These tools reduced decisional conflict and increased knowledge about treatment options for patients with depressive [8] and post-traumatic stress disorders [9]. One CDA facilitated shared decision-making (SDM) for patients with psychotic disorders [10]. To improve adoptation of CDAs in psychosis care, Lentis Psychiatric Institute developed a computerized CDA: Treatment E-Assist (TREAT) [11]. TREAT is the first CDA in psychosis care that combines routine outcome monitoring (ROM) data with current treatment guidelines and care standards to provide clinicians and patients with personalized evidence-based treatment recommendations. Care providers in the Northern-Netherlands use an extensive ROM-screening in psychosis care called the Pharmacotherapy Monitoring and Outcome Survey (ROM-PHAMOUS) [12]. Routine outcome data are ideally used to draft treatment plans during annual consultations. There is no formal procedure for integrating these data into daily clinical practice. As such the way information from ROM-PHAMOUS is directly used to guide treatment varies between institutions, teams and clinicians. Although ROM-PHAMOUS is effective in identifying care needs, these needs are not always met with appropriate care [13, 14]. TREAT was designed to bridge the gap between ROM-data and treatment choice by offering customized treatment recommendations to discuss during the annual treatment plan evaluations. TREAT is evaluated on its clinical effectiveness in improving care in a multicenter study.

### Research aim

The focus of this study is on the usage and evaluation of TREAT by clinicians. Three aims were formulated: 1) assess how clinicians use TREAT during consultations, 2) gain greater understanding in user acceptance by investigating how clinicians evaluated TREAT, and finally 3) collect information on how to improve the application for future use.

## Method

### Study setting and participants

During the trial, clinicians were asked to use TREAT, each with four different patients, during their annual treatment plan evaluations. In total, 33 clinicians enrolled in the trial of which 27 actually worked with the TREAT application. In-depth interviews were conducted with those clinicians who used TREAT with at least three different patients. In total, 13 clinicians who met this criterion were approached and agreed to participate. They worked in 11 different flexible assertive community treatment (FACT) teams of four mental healthcare institutions.

### Study design

**Fig. 1:**
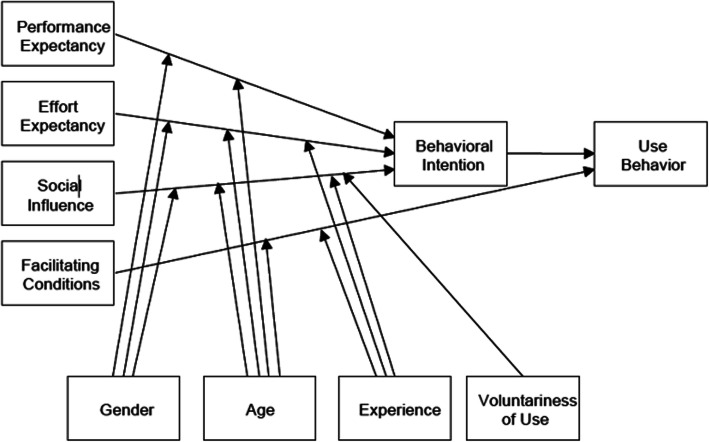
Unified theory of acceptance and use of technology

A descriptive qualitative design was used to gain insights into the experiences and attitudes of clinicians who worked with TREAT. The Unified Theory of Acceptance and Use of Technology (UTAUT, Fig. 1) [15] was used as a sensitizing theory to structure our semi-structured interview guide, as shown in additional file 1. This model is a reference to explore an individual’s intention to adopt a technological innovation in an organizational setting. In the UTAUT model performance expectancy, effort expectancy, social influence and facilitating conditions predict usage behavior [15]. These predictive factors are moderated by gender, age, experience (occupation and years working in psychosis care) and voluntariness of use [15]. Additional questions structured in accordance with our research aim were added to the interview guide (see additional file 1) such as “In what way did you use TREAT?” or “What was the effect of TREAT on your clinical encounters?”

### Data collection

All data was collected between July and September of 2019. Clinicians were invited through telephone calls to participate in this study and interviews were planned with those who were willing to participate. All interviews took place in the office of clinicians. The assessment started with a brief introduction to explain the goals of the interview and to sign the informed consent. Subsequently, the semi-structured interview was conducted by the first author (LR) based on the interview guideline. All questions were open and the researcher asked in-depth questions to further elucidate unclear or ambiguous information. The interviews lasted between 15 and 45 min. All interviews were digitally recorded and transcribed verbatim. Our goal was to identify as many relevant themes as possible. No new information or themes emerged after 13 interviews after which data saturation was assumed and data collection was stopped [16].

### Data analysis

We analyzed the data using an inductive thematic analysis approach [17]. With the use of the qualitative data analysis software of ATLAS Ti version 8.4, every transcript was coded line-by-line by the first author to identify patterns and gaps in the data. Two hundred seventy-three codes were identified. Using an inductive approach, the research team identified themes from the codes [18]. Finally, the research team discussed the themes on relevance until consensus was reached.

## Results

We interviewed 13 professionals: eight psychiatrists and five nurse practitioners. The average participant age was 49, seven were female and their average experience with working in psychosis care was 17 years (Table 1). Eight clinicians experienced the application as overall benefiting their clinical encounters, whereas five experienced no or even a negative impact on their daily clinical practice. The research group identified five recurrent themes in the interviews: 1) graphic representation, 2) guideline based treatment recommendations, 3) contextual factors, 4) effects on patients and 5) effects on shared decision-making. These themes were appraised differently by the respondents and provided new insights into the way TREAT was used during consultations and contained feedback that can be used to improve TREAT for future use.

**Table 1:** Clinician demographics

Clinician	Occupation	Age range	Years in psychosis care
**1**	Psychiatrist	61–65	23
**2**	Psychiatrist	41–45	3
**3**	Nurse practitioner	41–45	21
**4**	Psychiatrist	66–70	33
**5**	Psychiatrist	41–45	11
**6**	Nurse practitioner	41–45	20
**7**	Psychiatrist	56–60	23
**8**	Psychiatrist	36–40	5
**9**	Nurse practitioner	36–40	20
**10**	Psychiatrist	51–55	19
**11**	Nurse practitioner	61–65	15
**12**	Psychiatrist	41–45	15
**13**	Nurse practitioner	46–50	12

### Theme 1: Views on TREAT’s graphic representation

**Fig. 2:**
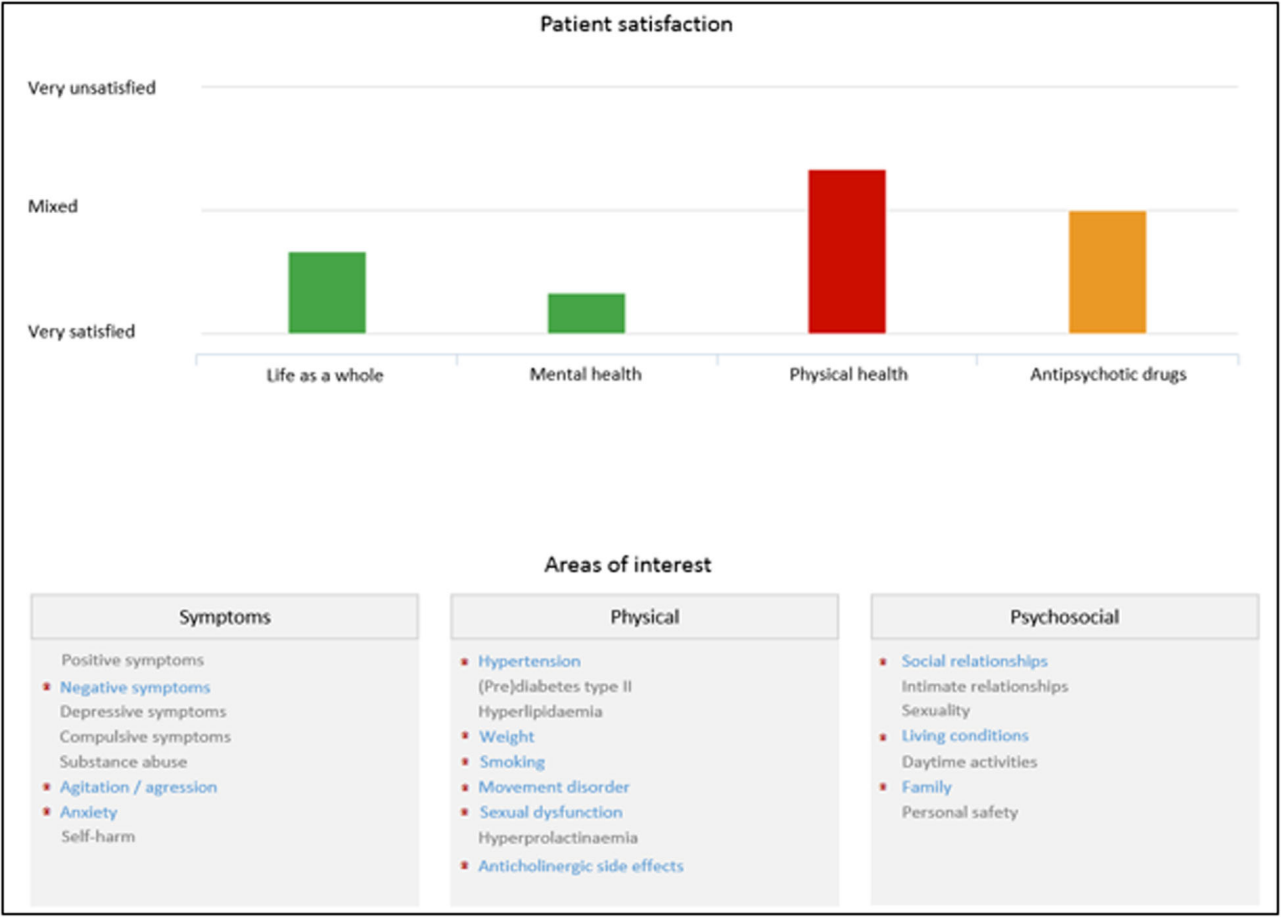
Example of the graphic data representation

Before the introduction of TREAT, ROM results were summarized in a letter to the clinicians and the general practitioner (i.e. ‘ROM-letter’). It contains a written description of the ROM results. The TREAT application presents ROM results graphically and structures it in three areas (symptoms, physical health and psychosocial wellbeing, Fig. 2). This representation was frequently discussed. The majority of the respondents indicated that, compared to the ROM-letter, TREAT reports were an improvement. Data is better structured and more appealing. The graphs made it easier to identify and interpret issues and the visualization improved the discussion with patients. One clinician noted:

*“It’s a really good instrument to interpret the ROM-results and to take action if needed. It makes things a lot easier. With the ROM-letter, “you had to figure out what should be discussed with the patient, and which matters were less important. With TREAT it’s much more obvious, so yeah, it’s much easier.”* [C1]

Another clinician emphasized the visualization of outliers in the results:

*“TREAT is very user-friendly and the graphs also make it very visual. People were able to really see the outliers in their results, which gives me the opportunity to specifically discuss them. It gives people guidance and support during the consult.”* [C8]

However, some clinicians indicated that TREAT added little value to their already structured routine:

*“Let me start by saying that our ROM-letter, which we have been using for years, has a clear overview of all ROM results. Therefore, I am already used to evaluate these results systematically with my patients. With TREAT this remains the same albeit in a different visual representation with graphs and treatment recommendations.”* [C12]

While some clinicians felt TREAT complicated their routine:

*“I always used the ROM-letter myself to check for any particularities, somehow there always seemed to be less than with TREAT. Now the focus is on many more areas, so you almost need to prepare ahead of time.”* [C5]

Overall, the representation of the ROM-results was well-appreciated by most clinicians and seen as an improvement compared to the previous ROM-letter. Graphic representation of the ROM-results made pressing issues in treatment more visible and therefore easier to discuss. Based on the UTAUT model, TREAT’s graphical representation positively affected the predictive factors of effort and performance expectancy.

### Theme 2: Views on TREAT’s treatment recommendations

**Fig. 3:**
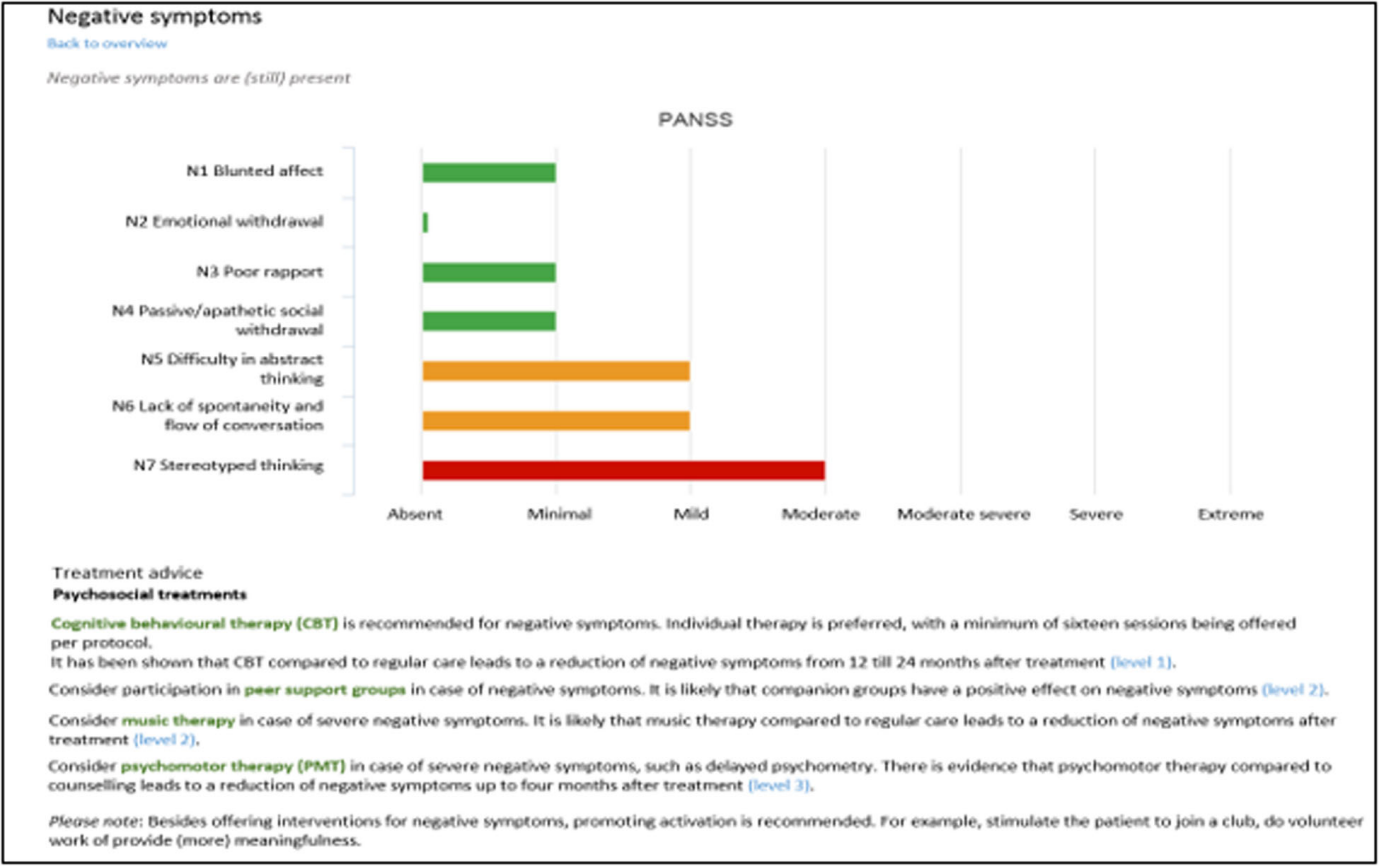
Example of treatment recommendations

TREAT offers several treatment recommendations (Fig. 3) for clinicians to consider with their patients. Some clinicians found these recommendations helpful:

*“That’s what I like about TREAT; you are not forced to follow for example a recommendation to start an anti-depressant in case of persistent negative symptoms. You just discuss it, like is this something you would prefer or not. Maybe you both decide to try something else. Either way the recommendation is still valid, it’s just not mandatory.”* [C6]

However, others found the recommendations bothersome or felt pressured:

*“I mean, I know it’s not mandatory to follow the recommendations, but it still feels that way. Sometimes, you’re just happy that somebody is using the medication you prescribe at all, and then you get the recommendation to switch the medication. TREAT seems to always tell you that it’s not good enough. It’s never good enough.”* [C13]

Even though the tone of the treatment recommendations was experienced in different ways, all clinicians agreed that the actual content of the recommendations was sound. However, opinions on the applicability varied. Several clinicians experienced the suggestions as generic and comprehensive, sometimes even too comprehensive. Not all recommendations were suited for the clinical complexity of the patient, or had already been tried before:

*“The treatment recommendations are sound but you always need to tailor them to a specific patient or circumstance and see if they still apply.... It’s difficult because sometimes certain recommendations from guidelines have already been tried or are not applicable anymore.”* [C2]

Although several clinicians raised the issue of utility, most of them had checked the utility and relevancy of the recommendations for each individual patient and found ways to incorporate them into their consultations. The recommendations were used to evaluate previous steps and to discuss and decide on current treatment plans, as this respondent explained:

*“TREAT is helpful in aligning treatment with the evidence-based recommendations. It can be used to start a conversation about treatment options and help explain why alternative treatment options might be more preferable.”* [C11]

Other clinicians used the recommendations to discuss possible future steps in the treatment process:

“*TREAT also provides information about possible future steps in treatment such as for example electroconvulsive therapy. If recommendations are presented on a screen it feels more natural to address it as an option. You can inform patients of different options in case the current treatment doesn’t work.”* [C8]

However, some clinicians did not see the recommendations as beneficial. They argued that they were well aware of the content of existing guidelines and therefore did not need an overview of the different guideline-recommended treatment options, as this respondent stated:

*“TREAT was not beneficial in reminding me of new things we could try for a specific problem. It’s not really a lack of knowledge I experience when drafting a treatment plan or when starting a new treatment.”* [C12]

Some respondents even experienced the recommendations as irritating:

“*I feel guidelines are necessary as a foundation but we can also assume they are well-known. To build a system just to beat people over the head with guidelines defeats its purpose. It irritates.”* [C10]

Overall, opinions on the recommendations varied. Some respondents actively used the recommendations during their consultations while others felt no need for guideline implementation. Multiple suggestions were made to shorten the text and to make recommendations more personalized. It is important to look for ways to improve these recommendations, as a perceived lack of utility could potentially prevent clinicians from working with TREAT after this clinical trial.

### Theme 3: Views on TREAT’s contextual factors

All clinicians agreed that TREAT was properly imbedded into the existing technical infrastructure of the electronic patient record. Therefore, the facilitating factor referred by the UTAUT model was perceived as optimal and enhanced TREAT’s use. Clinicians who experienced TREAT as benefiting their practice found it easier to incorporate the application into their routines. Teams that used a strict screening routine, organized processing of the screening data and structured scheduling of treatment plan evaluations, were most successful at implementing TREAT. Some teams used the opportunity of the TREAT study to improve their screening process and feedback procedure, as this clinician highlighted:

*“We chose to participate in the TREAT study and to make TREAT the driving force behind our evaluations and yearly screenings.”* [C9]

Getting used to a tool such as TREAT, even if ultimately intended as a time-saver, takes time [15]. Most teams indicated, however, that they simply did not have that time, as they were understaffed. Some clinicians indicated that the TREAT application made their consultations more time efficient. However, the majority experienced either no difference or reported increased consultation times. Most clinicians had to become familiar with TREAT and find ways to use it effectively during consultations:

*“You really need to work with it [TREAT] a few times because you can get questions for which you were not prepared or reminded of things you might have missed.”* [C7]

Apart from novelty, TREAT also increased consultations times by bringing up a larger array of topics for discussion:

*“I think my consultations became longer, because I noticed some time shortage. Therefore, you probably take or just need some more time to discuss all the results. It depends of course, on what ends up in TREAT. If someone has few problems you are quicker to discuss everything.”* [C12]

This is in part because the ROM-PHAMOUS screening is extensive and patients often experience issues in multiple areas. Furthermore, an incomplete screening was mentioned several times as a limiting factor. Clinicians sometimes chose not to use TREAT during a consultation because questionnaires were missing. In addition, more than half of the clinicians indicated that some of the recommended interventions were not part of the available treatment resources within their team. In some teams, nearly all of the recommended interventions were unavailable. Psychomotor therapy (PMT) was mentioned most frequently as a missing resource, followed by cognitive behavioral therapy (CBT), eye movement desensitization and reprocessing (EMDR) and individual placement and support (IPS). Clinicians generally ignored unavailable recommendations during consultations, thereby potentially decreasing TREAT’s efficacy. In some cases, TREAT motivated clinicians to recruit professionals for missing resources elsewhere in their organization:

*“I really feel a lot of the added value lies in the fact that we are used to recommending treatments we have available. TREAT reminds you of treatments you do not have available directly, so you can try and find those treatments elsewhere within the organization.” [C8]*

Compared to most CDAs in fields such as oncology, cardiology or orthopedics [2], psychiatry differs from settings in which CDAs have found mass adoption because care for patients in FACT teams is mostly integrated in long lasting recovery based processes [19]. Treatment decisions fit in an approach in which timing of interventions is important. Interventions should be available at various times throughout the treatment process. Moreover, psychotic illness is periodic and the decision-making process should match this process-based variability. It was mentioned several times that it is not always straightforward to turn treatment recommendations into behavioral changes for this patient group:

*“Most people have been in care for a long time and suffer from several disabilities. Sometimes you are able to initiate something new by putting in a lot of effort, but sometimes it just does not work because some patients have been doing things in a certain way for so long it’s difficult to motivate them to try things in a new way.”* [C7]

On the other hand, some clinicians actually used TREAT as a driving force to try new steps in treatment without postponing them:

*“I think it [TREAT] helps clinicians to stay closer to and be more professional in chronic treatment while remaining evidence-based without postponing the next step in treatments.”* [C8]

To summarize, structured and complete ROM screenings facilitate the use of TREAT. Consultations need to be strictly planned after screenings and might take more time. Missing treatment resources within teams can lower the efficacy of TREAT and hamper its implementation.

### Theme 4: Views on TREAT’s effects on patients

The effects of TREAT on individual patients were a recurring theme. The extent to which patients were engaged in the use of TREAT varied. In most cases, patients and clinicians sat together in front of a computer screen to review the TREAT report. However, some clinicians preferred to use the printed version. Overall respondents noticed that sharing information with TREAT did not work equally well for all patients. Some had cognitive problems and were easily overwhelmed by the complexity of the data presented in the application:

*“I noticed that if patients are not able to process a lot of information at the same time or if they are very much stuck in their own line of thinking, TREAT’s systemic approach doesn’t really work that well.”* [C2]

Most clinicians indicated that they did not notice significant changes in the therapeutic relation with their patients when using TREAT. However, some clinicians did:

*“We think that the traditional treatment relationship between patient and clinician is fundamentally changing, it is becoming more horizontal, not in every aspect but in many. That is where it is supposed to go. I really think TREAT can facilitate this because it increases commitment and a feeling of ownership.”* [C4]

Another clinician noticed a greater sense of ownership for patients while using TREAT:

*“It really has to do with ownership of the data. If I have a ROM-letter with a lot of text, it feels like I own the data. With TREAT there is a subtle nuance in how it feels, like you give the patient more ownership and make them the owner of the data.”* [C2]

Most clinicians viewed TREAT as an effective tool to engage in conversation with patients about specific areas of interest or suggested treatment recommendations:

*“You can show your patients the different treatment options during the consultation and explain the risks and benefits. I see it as a useful tool to engage in a conversation about the available treatment options.”* [C3]

It was often mentioned that TREAT prevents you from missing certain issues during consultations. This opens up the opportunity to discuss these issues with patients, as this respondent revealed:

*“Of course, that’s the beauty of this system. TREAT suggests things that you otherwise might have forgotten or wouldn’t have thought of. Sometimes it can be used to engage in conversation. For instance saying something like: “According to the guidelines, you would have to start with an antidepressant. What do you think? Oh, you don’t want another pill? Okay.” [C6]*

Several clinicians indicated that it became easier to discuss intimate topics because they were explicitly stated in TREAT. One respondent pointed out sexuality as an example:

*“For example sexuality. That is not something you would immediately discuss, I mean you should of course, so that is my fault, but with TREAT, it is explicitly stated. Also intimacy. It therefore brings itself up, which makes you talk about it. So that’s an improvement.”* [C4]

Some clinicians expressed concerns that TREAT focusses more on problems instead of strengths. Highlighting the positive trends and aspects of treatment was mentioned several times as a potential improvement. Most clinicians have a recovery-oriented view on patient care, which sometimes contradicted the alarming nature of TREAT as this respondent explained:

*“Our intention in our patient contact is to try to focus on recovery and strengths. However, TREAT draws the attention mostly to the negative points.”* [C13]

In a few cases, patients experienced TREAT as confrontational and it even scared some:

*“Sometimes I would notice a negative atmosphere, caused by the results and how they are displayed. That’s because it mostly highlights problems which pop up in red graphs. That scared some patients.”* [C12]

In sum, some clinicians noticed some patients did not respond well to TREAT because it confused or scared them. However, clinicians were able to use TREAT effectively during consultations with most of their patients. Important and sensitive issues became apparent and were therefore less likely to be forgotten which strengthened clinicians’ performance expectancy as referred to in the UTAUT model.

### Theme 5: Views on TREAT’s effects on shared decision-making

A majority of clinicians indicated that TREAT supported their clinical reasoning. It did not change the outcome of most treatment decisions, but improved the way these decisions were made. Even though clinicians held different opinions regarding the benefits of TREAT, nearly all of them agreed that it contributes to shared decision-making (SDM):

*“It [TREAT] did have a positive influence on shared decision-making. You have multiple options to choose from. That was most obvious with things like negative symptoms. You can tell someone music therapy or cognitive behavioral therapy is available, but scrolling through these options together makes it easier for patients to say: ‘that doesn’t suit me, but this is something I’d like to try'.”* [C8]

Another clinician provided a practical example of TREAT contributing to SDM during a consultation:

*“It [TREAT] improves your thinking. For example with a patient suffering from depression and a guilt delusion. For the delusion, it was recommended to start clozapine, but for the depression, the recommendation was to start a lithium addition. You explain and discuss these options. Eventually we both agreed to start with the depression protocol, before starting clozapine. We also agreed it was a mood congruent delusion. TREAT really helps to show things in this way.”* [C11]

In conclusion, although respondents have different opinions about the benefits and different aspects of TREAT, they all agree that the application facilitates shared decision-making. In total, five themes explain the use and evaluation of TREAT by clinicians (Table 2). In addition, all four predictors of the UTAUT model were positively evaluated by the majority of the respondents.

**Table 2:** Summary of results

Views on TREAT’s:	TREAT experienced as promoting	TREAT experienced as limiting
**1. Graphicrepresentation**	Improved representation versus ROM-letter	Successful feedback routines not in need of change
**2. Treatment recommendations**	Supported clinical reasoning and discussions with patients	Too generic, comprehensive or inapplicable
**3. Contextual factors**	Structured and complete screening routines	Time pressure and unavailable resources
**4. Effects on patients**	Sense of ownership and increased commitment	Overwhelming and difficult
**5. Effects on shared decision-making**	Facilitated shared decision-making	Clinical decisions often remained unchanged

## Discussion

To the best of our knowledge, this is the first qualitative study to examine the attitudes of clinicians working with a CDA in psychosis care. Clinicians were the primary focus of this study because they have to adopt TREAT for successful implementation. Clinicians reported both positive and negative experiences with the TREAT application. Two groups emerged: those who experienced TREAT as beneficial to their daily clinical practice and those who did not. Psychiatrists were divided, while nurse practitioners held the most positive attitudes. Nurse practitioners were more willing to change their normal consultation routines and use the guideline-based treatment recommendations. Age and work experience in psychosis care, did not influence the use nor evaluation of TREAT. Thematic analysis revealed five recurrent themes that provided insights into how TREAT was used and perceived.

Many respondents mentioned time pressure as a prevalent issue. TREAT did not decrease the average consultation time. This was mostly due to comprehensive coverage of topics and because clinicians had to become familiar with the application. In general the consultation time increased but at the same time more topics were discussed, oftentimes in a more efficient and collaborative way. The graphical user interface was experienced as an improvement over the existing text-based report letter provided by the routine outcome monitoring (ROM) system. For most clinicians it became easier to integrate the feedback of ROM-results into their consultations with patients. This is an important finding, as previous research has shown that CDAs that are difficult to integrate in existing care processes are not used by clinicians after the experimental introduction [4].

In contrast with the positive responses on the visual presentation of the individual ROM data, clinicians held different opinions about the guideline-based treatment recommendations. Some respondents indicated to be well aware of existing guidelines and questioned the usefulness of TREAT’s recommendations for daily clinical practice. Other clinicians did use the recommendations to support their clinical reasoning and to discuss the suggested interventions with their patients. Applicability was an issue for some of the recommendations, as they were sometimes experienced as too generic, required missing resources, or had already been suggested or tried before by patients. Only some clinicians mentioned interventions or treatments that were started following treatment recommendations of TREAT. Cognitive behavioral therapy (CBT) and eye movement desensitization and reprocessing (EMDR) for example, were often recommended but in several teams unavailable due to absence of a specialized psychologist. This is in line with a regional psychosis care assessment in The Netherlands, which revealed limited availability for several guideline-based interventions [20]. Unavailability of resources has to be addressed in order to successfully implement tools such as TREAT. The availability of recommended interventions is a prerequisite for the efficacy of any CDA [1, 4].

Although clinicians were divided about the benefits of the treatment recommendations, they all agreed that TREAT contributed to more shared decision-making (SDM). This is an important finding, as SDM in psychosis care is considered desirable, yet difficult to achieve [21]. Making shared decisions can increase treatment adherence and bolster empowerment of people with psychotic illness [22, 23]. SDM tries to change the traditional power asymmetry between patients and clinicians [24]. The treatment recommendations and the graphic representation of the ROM-results can contribute to SDM by strengthening the exchange of information and the decisional position of patients. Some clinicians noticed more commitment from their patients during consultations and a stronger sense of ownership of their ROM-data, when using TREAT. It has to be noted that not all patients responded equally well to the application according to clinicians, with some even having negative responses. Concerns were also expressed about TREAT in relation to personal recovery. Some clinicians stated that the focus might still be too much on the symptoms, burdens and problems, instead of strengths and the opportunities for support and treatment. However, increasing SDM, autonomy and a sense of ownership over the data during consultations, makes TREAT compatible with personal recovery-oriented care [25].

We also utilized the Unified Theory of Acceptance and Use of Technology (UTAUT) as presented in Fig. 1 to summarize our findings. Most interviewed clinicians perceived TREAT as beneficial, user-friendly, easy to use and experienced minimal burden. Therefore, the predictive factors of performance and effort expectancy were generally positively evaluated. There was little social influence because clinicians participated by their own choice. They were often the only ones in their team to join the study and experienced no external pressures to work with TREAT. All respondents agreed that the facilitating conditions for implementation of the application into the existing technical infrastructure and care processes were met. Although no statistical generalizations can be made from these findings, all predictive factors of the UTAUT model were positively evaluated by clinicians.

### Strengths and limitations

Some limitations of this study need to be addressed. One main limitation is a possible selection bias. We emphasized the importance of clinicians with skeptical attitudes towards CDAs in general and TREAT in particular while recruiting participants for this study. However, an oversampling of clinicians with a more favorable attitude cannot be ruled out. Furthermore, we only interviewed clinicians that worked with the application on at least three different occasions. We were interested in their opinions because they had enough experience to evaluate TREAT and help us understand the way in which the application was used. Clinicians that worked with TREAT only once or twice filled out a short anonymous questionnaire to assess what complications they faced in completing the intended four measurements. The most common reasons were clinicians discontinuing their work within the team or organization during the trial, consulting insufficient patients that met the inclusion criteria and logistical issues with planning and carrying out measurements. Although not specifically mentioned in the anonymous questionnaire, we cannot rule out the possibility that some clinicians who were not included in this study had stopped using TREAT after trying it once or twice because of a perceived lack of benefit. The main strength of this study is that we reached data saturation with a diverse group of respondents. Both male and female psychiatrists and nurse practitioners from different ages and with varying years of experience in psychosis care were interviewed. No psychologist participated because they are underrepresented in psychosis care and are less involved with the annual treatment plan evaluations. Clinicians were recruited from teams in four different institutions from both urban and rural areas. The diversity of respondents supports the generalizability of our results.

### Future research and improvements

This study provides insights into the use and evaluation of TREAT by clinicians. Some questions about the effects of TREAT on daily clinical practice remain unanswered. Furthermore, several improvements were suggested for future use. Nearly all clinicians perceived more SDM when working with TREAT. This requires additional quantitative assessment. Furthermore, it is important to investigate the perspectives of patients because we do not known how they experience CDAs in general and in psychosis care in particular. Our current trial measures patients’ decisional conflict to assess whether TREAT can facilitate SDM during consultations. Patients’ ratings of these consultations are also included. According to clinicians, not all patients responded equally well to TREAT, because the focus is mainly on problems and less on the improvements in treatment. For future development, clinicians often recommended incorporating ROM-data from previous years to visualize positive health trends of patients in treatment. They also suggested increasing the interactivity of TREAT. For example, by asking patients to prioritize areas of interest or by adding small scripts with relevant questions for identified problems. Furthermore, multiple clinicians requested a similar application for other mental illnesses such as depressive, borderline or bipolar disorders. It is worthwhile to explore new ways of collecting data, for example with the experienced sampling method or by utilizing smart devices [26]. Moreover, newly developed models and guidelines for the deployment of machine learning and artificial intelligence in psychiatry, could improve new CDAs [27]. A continuously growing body of evidence could provide future designers with guidelines for development and implementation of these tools.

## Conclusions

This article describes the use and evaluation by clinicians of a computerized clinical decision aid in the treatment of people with psychotic disorders. Most clinicians experienced TREAT as easy to use and beneficial to their consultations. The structured visual representation of ROM-results was generally well-appreciated and provided cues to discuss treatment planning with patients. The guideline based treatment recommendations supported clinical reasoning but did not seem to change most treatment decisions. The delivery and applicability of the recommendations and the availability of the recommended interventions need improvement for successful implementation. Most patients seemed to appreciate TREAT but not all, according to clinicians. The observation that TREAT facilitates shared decision-making is promising but requires further quantitative assessment. Future research should also focus on patients’ perspectives to investigate which patients respond best to CDAs in psychosis care. All four predictors of the Unified Theory of Acceptance and Use of Technology were positively evaluated by the majority of clinicians. The full impact of TREAT on daily clinical practice still has to be assessed in the ongoing clinical trial and in future research.

## Supplementary information

**Additional file 1.** Guide for the clinicians interview. The interview guide used for the in-depth interviews.

## Data Availability

The authors approve on making the datasets used in this study available after anonymization, when requested to the corresponding author upon reasonable request.

## Abbreviations

CDAs: Clinical decision aids
FACT: Functional assertive community treatment
ROM: Routine outcome monitoring
ROM-PHAMOUS: Routine outcome monitoring - pharmacotherapy monitoring and outcome survey
SDM: Shared decision-making
TREAT: TReatment-E-AssisT
UTAUT: Unified theory of acceptance and use of technology model
