# Supplementary figures and images for: Qualitative analysis of clinicians’ perspectives on the use of a computerized decision aid in the treatment of psychotic disorders
Source: BMC Med Inform Decis Mak. 2020 Sep 17;20:234. doi: 10.1186/s12911-020-01251-6 (PMC7499839; doi:10.1186/s12911-020-01251-6)

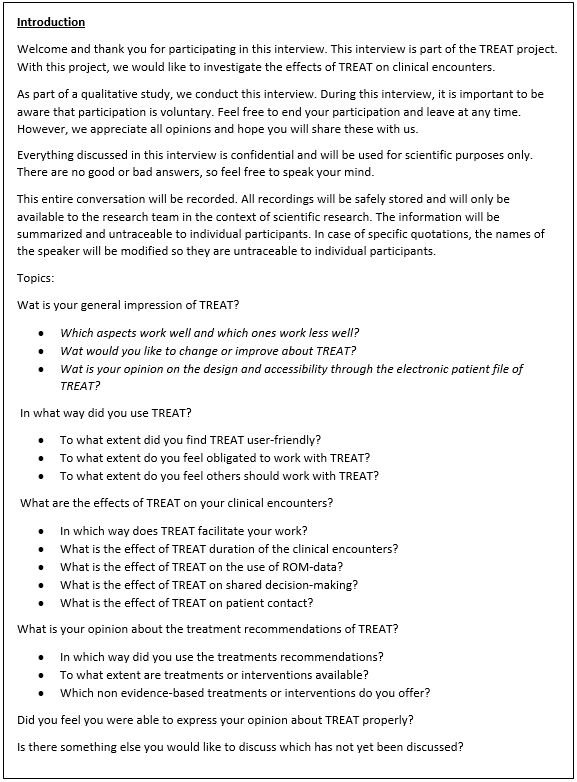

Supplement: Supplementary file 1 — Additional file 1. Guide for the clinicians interview. The interview guide used for the in-depth interviews. [file 12911_2020_1251_MOESM1_ESM.png]
